# Feto-maternal outcomes of hypertensive disorders of pregnancy in Yekatit-12 Teaching Hospital, Addis Ababa: a retrospective study

**DOI:** 10.1186/s12872-020-01399-z

**Published:** 2020-04-15

**Authors:** Mekoya D. Mengistu, Tilahun Kuma

**Affiliations:** 1 Department of Physiology, School of Medicine, Addis Ababa University, Addis Ababa, Ethiopia; 2 Department of Internal Medicine, Yekatit-12 Hospital Medical College, Addis Ababa, Ethiopia; 3 Department of Gynecology and Obstetrics, Yekatit-12 Hospital Medical College, Addis Ababa, Ethiopia

**Keywords:** Hypertensive disorders of pregnancy, Feto-maternal outcomes, Yekatit-12 hospital

## Abstract

**Background:**

In resource poor countries, hypertensive disorders of pregnancy are common and form one of the deadly triads, along with hemorrhage and infection, which contribute greatly to maternal and fetal jeopardy.

**Methods:**

The aim of this study was to assess the prevalence of hypertensive disorders of pregnancy, and determine the effects of hypertensive disorders of pregnancy on the feto-maternal outcomes. It was a descriptive, cross-sectional, retrospective study on randomly selected 615 women who attended delivery at Yekatit-12 Teaching Hospital from 1st of July 2017 -1st of Jan 2018. Data was analyzed using SPSS version 20 software. Descriptive statistics were used to calculate rates. Chi-square statistics were used to estimate the associations among selected predictor variables. A *p*-value < 0.05 was taken as statistically significant.

**Results:**

Out of the 615 study population, the prevalence of hypertensive disorders of pregnancy was found to be 25.4%, of which the majority (52.5%) was severe pre-eclampsia. Eclampsia accounted for 2.6%, and superimposed pre-eclampsia was 2.6%. The rate of severe pre-eclampsia with HELLP syndrome was 7.1% of all mothers with the hypertensive disorders. The majority of mothers with hypertensive disorders (59.6%) had age range of 25–34 years. About 46% of mothers required interventions to terminate the pregnancy either by cesarean section (42.3%) or instrumental deliveries (3.7%) due to conditions related to Hypertensive disorders. The rate of preterm, low birth weight, and low Apgar at 1st and 5^th^minutes accounted for 29.5, 24.4, 22.4 and 16.7% of neonates born to mothers with hypertensive disorders, respectively. Over 10.9% of neonates required resuscitation and 11.5% NICU referral. The rate of still birth was 3.8%.

**Conclusion:**

The prevalence of hypertensive disorders of pregnancy is high in the study area and complicates maternal and fetal outcomes of the pregnancy. To deter its detrimental effects both on fetal and maternal outcomes of pregnancy, antenatal surveillance should be expanded to enable early detection, stringent follow-up and timely intervention in severely affected pregnancies.

## Background

Hypertensive disorders of pregnancy (HDP) are common medical complications of pregnancy and form one of the deadly triad, along with hemorrhage and infection, contributing greatly to maternal, fetal and neonatal morbidity and mortality [1]. The hypertensive disorders in pregnancy includes chronic hypertension and the group of hypertensive disorders unique to pregnancy including gestational hypertension, preeclampsia and eclampsia. Approximately 30% of hypertensive disorders in pregnancy are due to chronic hypertension, and 70% are due to pregnancy induced hypertension [2]. ACOG estimated that hypertensive disorders complicate about 12–22% of pregnancies [3] and it has diverse risk factors [4–7]. In Ethiopia the prevalence of HDP and their effects on feto-maternal outcomes are obscure due to disproportionately low institutional delivery and also limited studies conducted in this important area so far. These limited studies indicate that the HDP contributed to a very significant maternal and neonatal disease burden [8]. The HDP complicated wide ranges of institutional deliveries from 5 to 8.5% [9, 10] to 18.25–25.1% [11, 12].

The spectrum of HDP ranges from mildly elevated blood pressures with minimal clinical significance to severe hypertension with multi-organ dysfunction [2]. Gestational hypertension can be defined as a systolic blood pressure (SBP) of ≥140 mmHg and/or a diastolic blood pressure (DBP) of ≥90 mmHg on at least two occasions at least 6 h apart after the 20th week of gestation in women known to be normotensive before 20 weeks’ gestation [13]. Preeclampsia is primarily defined as gestational hypertension accompanied with proteinuria (300 mg or more per 24-h period). If the 24-h urine collection is not available, proteinuria can be defined as a concentration of protein ≥30 mg/dL (≥1+ on dipstick) in at least two random urine samples collected at least 6 h apart [3, 4, 14]. In the absence of proteinuria, preeclampsia should be considered when gestational hypertension is associated with end-organ damages such as persistent cerebral symptoms, epigastric or right upper quadrant pain with nausea-vomiting, or thrombocytopenia and abnormal liver enzymes [4]. Preeclampsia is considered severe in the presence of one or more of the end-organ dysfunctions [3, 4]. A particularly severe form of preeclampsia is the HELLP syndrome, which is an acronym for three major abnormalities; hemolysis, elevated liver enzymes, and low platelet count [2]. The onset of convulsions in a woman with preeclampsia that cannot be attributed to other causes is termed eclampsia. The seizures are generalized and may appear before, during, or after labor [6].

The outcomes of pregnancy complicated by hypertensive disorders range from uneventful pregnancy in women with chronic but controlled hypertension to death in cases of preeclampsia-eclampsia [13]. The feto-maternal outcomes of hypertensive disorders of pregnancy are affected by multiple factors. These embrace but are not limited to gestational age at onset, severity of disease, the presence of multifetal gestation, and the presence of co-morbid conditions including diabetes mellitus, renal disease, thrombophilia, or preexisting hypertension [4, 13, 15]. WHO estimates that the incidence of preeclampsia is 7 times higher in low- and middle-income countries than in high-income countries, and the risk of a woman in a low-income country dying of pre-eclampsia/eclampsia is 300 times that of a woman in a high-income country [16]. The risk of death due to preeclamptic disease increases when eclampsia supervenes on the clinical picture. Pre-eclampsia/eclampsia is responsible for an estimated 16% of global maternal mortality annually [17].

The perinatal mortality and morbidities such as low birth weight, preterm, SGA, IUGR, need for resuscitation as well as NICU admission are substantially increased in women with severe preeclampsia [13, 18–21]. The high perinatal mortality in women with HDP is mainly due to premature delivery and growth restriction [20]. The multi-country survey of WHO has shown that there were about 3- and 5-fold increased risk of perinatal death in women with preeclampsia and eclampsia, respectively, as compared to women with no preeclampsia/eclampsia [22]. The majority of perinatal deaths due to complications of HDP have occurred in the low and middle income countries [23]. In Ethiopia, there are few hospital based reports [10, 23, 24] on the maternal and fetal outcomes of hypertensive disorders of pregnancy and they may not represent the larger population due to the low institutional delivery rate [25]. The objectives of this study were to assess the prevalence of hypertensive disorders of pregnancy, and determine its common feto-maternal outcomes.

## Methods

### Study design and setting

It was a descriptive, cross-sectional, retrospective study using data from the hospital records of pregnant women who delivered at Yekatit-12 Teaching Hospital from 1st of July 2017 up to 1st of Jan 2018. The study was conducted at the Gynecology and Obstetrics department of Yekatit-12 Teaching Hospital which is one of the Government referal hospitals in Addis Ababa, the capital of Ethiopia. The Hospital has 16 Health Centers with a total of about 1.5million population in its catchment area. The health centers in the catchment area are mandated to refer the risky pregnancies including those with hypertensive disorders of pregnancy to this Hospital based on the referral network system designed recently by the Ethiopian government as part of the strategies to decrease the existing high maternal and perinatal mortality and morbidity. The referral network system is coordinated by liaison office and Task forces which are directly over sighted by the city Health Bureau and the Ministry of Health. Therefore, the Gynecology and Obstetrics department is overwhelmed by gravid women referred from the Health centers in the catchment area, women already enrolled to have their ANC follow up at the hospital based on their bad obstetric history, and also women coming with referral from the nearby rural areas and other Hospitals.

### Study population

This study included all eligible women admitted to and delivered at the study hospital from 1st of July 2017 up to 1st of Jan 2018. A total of 615 samples from all women who delivered in the institution during the specified period were included with 100 subjects randomly selected from each month of the study period. The exclusion criteria include women who were transferred to other hospitals after being admitted to the study hospital, lost or incomplete data, or died on arrival before adequate diagnosis was made.

### Data variables for the study

In order to assess the maternal and fetal outcomes of hypertensive disorders of pregnancy, data was obtained from the medical records of the study subjects. The maternal parameters obtained included; chief complaints, maternal age, maternal vital sign, parity, gestational age, number of fetuses, other maternal risk factors for HDP (such as previous history of similar illness, diabetes mellitus, chronic kidney disease), Highest systolic and diastolic blood pressure recorded, type of HDP, onset of HDP, severity symptoms of HDP, type of anticonvulsant and antihypertensive given. Furthermore, onsets of labor, mode of deliveries, as well as obstetric complications were obtained. Laboratory findings included; proteinuria, hemoglobin, platelet count, serum creatinine, BUN, serum uric acid level, and serum liver enzyme levels. Neonatal outcome parameters obtained included; gestational age, birth outcome (alive or not), birth weight, APGAR score at 1st, and 5th minutes, need for resuscitation, and need for NICU admission.

### Quality control and data collection procedures

In order to ensure the accuracy, completeness, and comparability of data, the medical records of the study subjects were meticulously evaluated by the investigators. First the hospital registration numbers of the study subjects were obtained from the delivery registry logbook. Using these registration numbers the chart of each patient was retrieved from the archives. The ANC chart of women with follow-up at the hospital, referral chart of those referred from other health facilities, admission chart for women who were admitted to Maternity ward for inpatient follow up, admission chart to the labor ward for all women and their postnatal profile were thoroughly evaluated. Data collection was made by the pretested check lists to document all the pertinent profiles of the study subjects by the investigators themselves.

### Data processing and statistical analysis

After checking for completeness, data was coded, entered, and analyzed using SPSS version 20 software. Descriptive statistics was used to calculate rates. Chi-square was used to estimate the associations between selected predictor variables. A *p*-value < 0.05 was taken as statistically significant.

### Ethical consideration

Ethical clearance was obtained from institutional review board of Yekatit-12 Teaching Hospital. Since it was a retrospective study, written consent could not be obtained. Anonymity of the patient profile was upheld.

## Results

### Demographic and clinical characteristics of the study population

Data was collected from 615 samples randomly taken from all deliveries attended from 1st of July 2017 up to 1st of Jan 2018 at Yekatit-12 Teaching Hospital. The maternal age of the study population ranged from 14 years up to 42 years. About 60% of maternal age was in the range of 25 to 34 years (Table 1). However, only about 7% of mothers had at least 35 years of age. Primiparity accounted over half of the mothers and grand multiparity constituted almost less than 1% of the mothers. The rate of twin pregnancy in the study population was 3.6%. The majority of mothers had term pregnancy (78%) with preterm and very preterm both accounted for about 15% of the total, and the remaining being post term.

**Table 1:** Maternal and perinatal outcomes of the hypertensive disorders of pregnancy

Perinatal outcomes	Types of hypertension(156(25.36%))					
	Gestational hypertension 40 (25.6%)	Mild preeclampsia 28 (17.9%)	Severe Preeclampsia ⊕ 82 (52.56%)	Eclampsia 4 (2.6%)	Normotensive 459(74.6%)	***P***-value
**Gestational age**						
**< 34 weeks**	0	2(7.1%)	13(15.8%)	1(25%)	18(3.9%)	< 0.001*
**34-36 weeks**	5(12.5%)	2(7.1%)	22(26.8%)	1(25%)	30(6.5%)	
**≥ 37 week**	35(87.5%)	24(87.1%)	47(57.3%)	2(50%)	411(89.5%)	
**Birth weight**						
**< 1500 g**	0	1(3.5%)	10(12.2%)	0	8(1.7%)	< 0.001*
**1500-2499 g**	2(5.0%)	3(10.7%)	19(23.2%)	3(75%)	52(11.3%)	
**≥ 2500 g**	38(95%)	24(85.7%)	53(64.6%)	1(25%)	399(86.9%)	
**APGAR at 1st minute**						
**< 7**	3(7.5%)	7(25%)	23(28%)	2(50%)	61(13.3%)	0.001*
**≥ 7**	33(82.5%)	21(75%)	59(72%)	2(50%)	398(86.7%)	
**APGAR at 5th minute**						
**< 7**	1(2.5%)	4(14.2%)	19(23.2%)	2(50%)	26(5.6%)	< 0.001*
**≥ 7**	39(97.5%)	24(85.7%)	63(76.8%)	2(50%)	433(94.4%)	
**Birth outcome**						
**Still birth**	1(2.5%)	1(3.6%)	2(2.4%)	0	12(2.6%)	0.65
**Alive**	39(98.5%)	27(96.4%)	80(97.6%)	4(100%)	447(97.4%)	
**Neonatal complication**						
**No complication**	40(100%)	22(78.6%)	59(72%)	2(50%)	443(96.5%)	< 0.001*
**IUGR**^**a**^	0	1(3.57%)	10(12.2%)	2(50%)	0	
**Need for resuscitation**	0	3(10.7%)	18(21.9%)	2(50%)	15(3.2%)	
**Need for NICU**^**b**^	0	2(7.1%)	14(17.1%)	2(50%)	14(3.0%)	
**Maternal outcome**						
**Maternal age**						
**≤ 24 yrs**	16(40%)	6(21.4%)	26(31.7%)	0	165(35.9%)	0.157
**25-34 yrs**	21(52.5%)	20(71.4%)	47(57.3%)	4(100%)	263(57.3%)	
**≥ 35 yrs**	3(7.5%)	2(7.1%)	9(10.97%)	0	31(6.75%)	
**Mode of delivery**						
**vaginal**	23(57.5%)	13(46.4)	44(53.6%)	1(25%)	300(65.3%)	0.009*
**Instrumental**	1(2.5%)	3(10.7%)	2(2.4%)	0	36(7.8%)	
**cesarean**	12(30.0%)	12(42.8%)	35(42.67%)	3(75%)	123(26.7%)	

⊕ SPE, PE with HELLP and super imposed PE combined

^a^Intrauterine growth restriction

^b^Neonatal intensive care unit

*P-value < 0.05

Over 62% of mothers had spontaneous vaginal delivery, and instrumental delivery with vacuum and forceps accounted for additional 3 and 4% of deliveries, respectively. Episiotomy was done for 69% of mothers to facilitate their vaginal deliveries. Cesarean section was done to 30.7% of pregnancies for various obstetric indications. Most of the patient had multiple indications for cesarean deliveries. Preclampsia-Eclampsia was implicated in over 15% of the cesarean sections whereas previous uterine scar was implicated in about 25% of the cesarean indications. Non-reassuring fetal heart pattern (NRFHRP) was associated with about 20% of the total cesarean indications with half of the types of NRFHRP was persistent fetal bradycardia. The majority of neonates (79.75%) have birth weight in the range of normal birth weight whereas over 16% of neonates had birth weight of less than 2500 g. APGAR score less than 7 in the first minute accounted for 15.5% of neonates and APGAR score less than 7 in the 5th minutes was 8.4%.

Out of the 615 population studied, the prevalence of hypertensive disorders of pregnancy was found to be 25.4% (156), of which the majority (52.5%) was severe pre-eclampsia (Table. 1). The incidence of superimposed pre-eclampsia was 2.6% and eclampsia accounted for only 2.6%. The rate of severe pre-eclampsia with HELLP syndrome was 7.1% of all HDP. The majority of mothers with HDP (59.6%) had age range of 25–34 years and only 9% had age of at least 35 years. The association of maternal age to the severity of hypertension was not statistically significant (*p* = 0.441). Over one half (55.8%) of mothers with hypertensive disorders were primipara. The rate of twins’ pregnancy constituted 3.2% of mothers with hypertensive disorders with the remaining being singleton pregnancies.

### Maternal outcomes of hypertensive disorders of pregnancy

**Fig. 1:**
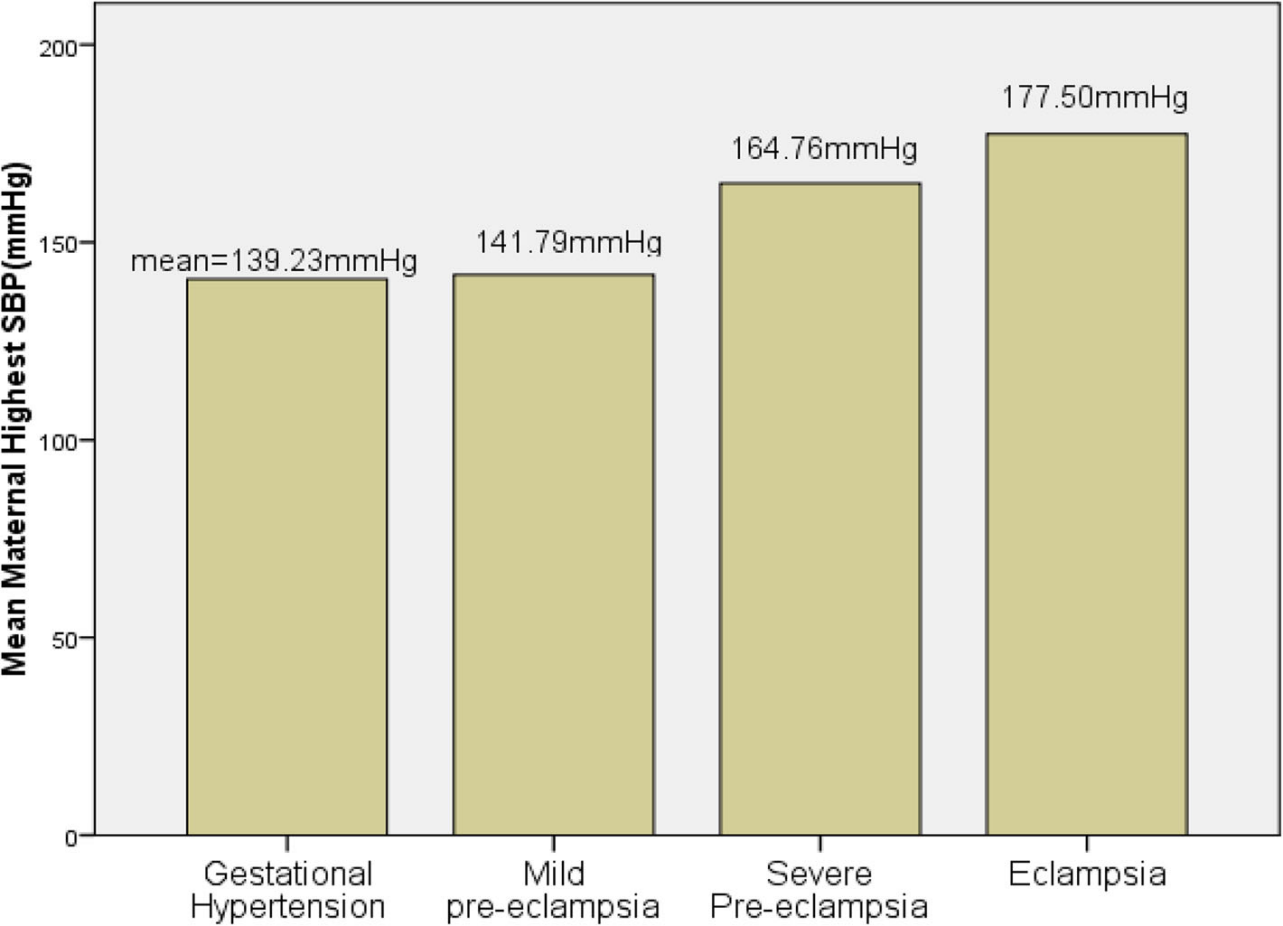
Bar graph of mean maternal systolic blood pressure in different classes of hypertensive diseases of pregnancy (*P* < 0.001)

**Fig. 2:**
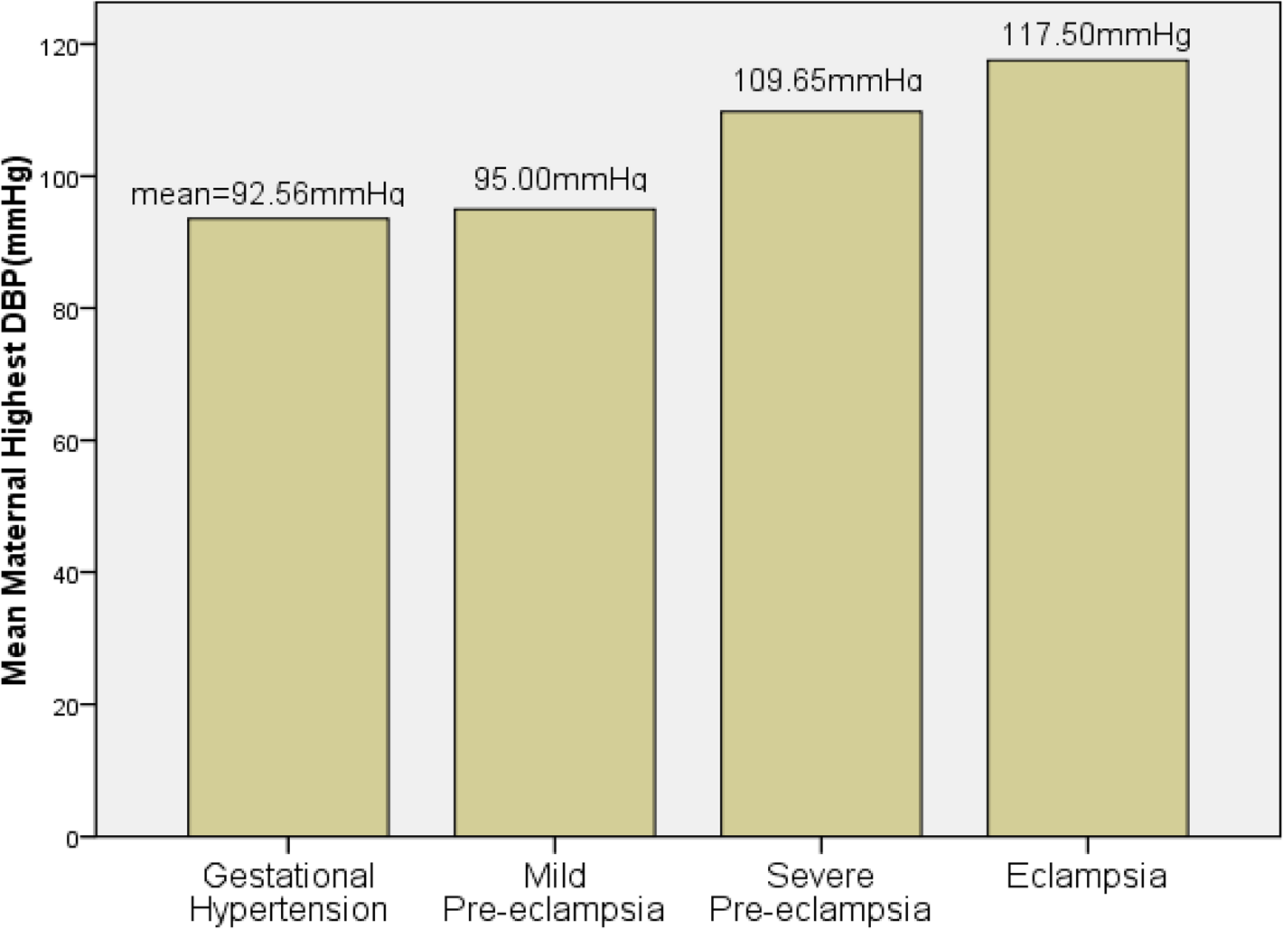
Bar graph of mean maternal diastolic blood pressure in different classes of hypertensive diseases of pregnancy (*p* < 0.001)

Vaginal delivery accounted for 53.8% of the mode of deliveries in all mothers with HDP. However, the rate of cesarean section contributed for 42.3% of deliveries. There were no destructive deliveries but instrumental delivery was 3.8% with forceps and vacuum had equal share. There was statistically significant association between severity of hypertensive disorders to the mode of deliveries with the risk of cesarean delivery increased with the severity of HDP (*p* = 0.009). Headache resistant to ordinary analgesics was among the chief complaints in 54% of mothers with severe pre-eclampsia and eclampsia. Similarly, blurring of vision was presented as part of the chief complaints in 39.4% of mothers with severe pre-eclampsia and eclampsia. All eclamptic mothers had generalized tonic-clonic seizure coupled with loss of consciousness as a chief complaint at presentation and had preceding history of headache and blurring of vision. About one fifth of mothers with severe pre-eclampsia and eclampsia developed acute renal failure (AKI) manifested by the creatinine level of at least 1.2 mg/dl (Cr ≥ 1.2 mg/dl) (Table 2). Thrombocytopenia (platelet< 100,000/mm^3^) was observed in 13.5% of mothers with severe pre-eclampsia and eclampsia. Over 88% of mothers with severe pre-eclampsia required hydralazine for immediate control of hypertension as did to all eclamptic mothers. About 92% of severe pre-eclamptic mothers who were given hydralazine also required another antihypertensive agent; mostly methyl-dopa for further stabilization of the blood pressure. All mothers with severe pre-eclampsia and eclampsia as well as 38.7% of mothers with mild pre-eclampsia were given MgSo4 for seizure preventive prophylaxis. The mean of systolic blood pressure and diastolic blood pressure in mothers with Gestational hypertension, Mild pre-eclampsia, Severe-eclampsia and Eclampsia were shown in the Figs. 1 and 2 below.

**Table 2:** Maternal effects of HDP: clinical conditions of severity features

Clinical manifestations	Mothers with Severe preeclampsia^a^ and Eclampsia (***n*** = 82)	All Mothers with HDP (***n*** = 156)
**Headache**	**48 (58.5%)**	**48 (30.8%)**
**Blurring of vision**	**35 (42.7%)**	**35 (22.5%)**
**Epigastric pain**	**9 (10.9%)**	**9 (5.8%)**
**Abruptio placentae**	**2 (2.4%)**	**2 (1.3%)**
**Seizure and Loss of consciousness**	**4 (4.87%)**	**4(2.6%)**
**AKI (cr ≥ 1.2 mg/dl)**	**17 (19.1%)**	**17 (10.9%)**
**Thrombocytopenia (plat < 100 × 10**^**3**^**)**	**12 (14.6%)**	**12(7.7%)**

^a^SPE, PE with HELLP and super imposed PE combined

### Perinatal outcomes of hypertensive disorders of pregnancy

The rates of very preterm and pre-term born to the mothers with HDP were 10.3, and 19.2%, respectively. Over 70% of women with HDP had GA of at least 37 weeks. There was statistically significant association between the severity of hypertensive disorders and prematurity (*p* < 0.001). Among the total hypertensive mothers, 7%(11) of them delivered babies with extremely low birth weight whereas the rate of low birth weight was 17.3% (27) with the cumulative rate of babies with birth weight less than 2500 g was 24.5%. There was also significant association between severity of hypertension with low birth weight (*p* < 0.001). The rate of Apgar score of less than 7 at first minute was 22.4% among all neonates delivered from hypertensive mothers. On the other hand, the rate of low Apgar score (less than 7) at fifth minute was 16.7% among all neonates delivered from mothers with hypertensive disorders. Compared with neonates of normotensive mothers with depressed Apgar score at first minute(13.3%) and fifth minutes ((5.6%), the depressed Apgar score was much higher among neonates delivered from mothers with mild pre-eclampsia (Apgar at first minute (25%) and fifth minutes(14.3%)), severe pre-eclampsia (Apgar at first minute (28.0%) and fifth minutes(23.2%)) and eclampsia ((Apgar at first minute (50%) and fifth minutes(50%)). This association of depressed Apgar sore with severity of maternal hypertensive disorders was statistically significant both at first minute (*p* = 0.007) and fifth minutes (*p* < 0.001).

Among neonates of eclamptic mothers, 50% of them required resuscitational support and NICU referral. Similarly, 12.2% (10) of neonates born to the mothers with severe pre-eclampsia were complicated with IUGR, 21.9% (18) needed the resucitational support, and 17% (14) needed referral to NICU. However, among normotensive mothers only 3.2% (15) of neonates were resuscitated and only 3.0% (14) neonates were referred to NICU. This increased likelihood of neonatal complications with increased severity of hypertension was statistically significant (*p* < 0.001). Nevertheless, there was no statistically significant increase in the rate of still birth among hypertensive mothers compared to the normotensive counter parts (*p* > 0.05).

## Discussion

Hypertensive disorders of pregnancy (HDP) are common and form one of the deadly triads, along with hemorrhage and infection, which contribute greatly to maternal morbidity and mortality, fetal, and neonatal jeopardy [1]. In our study, the prevalence of hypertensive disorders of pregnancy was 25.4%, of which severe pre-eclampsia was 52.5% and eclampsia accounted for 2.6% of the total cases of hypertensive disorders. The present prevalence is comparable to some of the previous studies in Ethiopa; 18.25% of preclampsia in Arbaminch [11] and 25.1% in Derashie, SNNP [12] and also findings in other countries [26, 27]. However, it is higher than some of the overall global reports [13, 16] as well as some studies in Ethiopia; 2.4% at Mettu Referral Hospital [24], 5.7%in Debrebrhan Referral Hospital [28], and 8.5% at Jimma Specialized Hospital [10]. The exaggerated prevalence of HDP in the current study is not surprising as the Hospital is a referral center receiving complicated pregnancies from 16 Health centers with catchment area of over 1.5 million populations as well as from other hospitals. Therefore, the current prevalence might be the effect of the aggregate rates from a number of primary care facilities in the catchment area with large denominator population. Studies also show that the increased prevalence is common in centers that serve as a referral medical facility for an extended number of primary care units [5]**.** The low socioeconomic status, young age, primiparity and urban residency are known risk factors for the development of preeclampsia [29] and could also be another contributing factors for increased prevalence of HDP in the present study. As the study was a retrospective type, measurement bias and errors could also have played a role. Severe pre-eclampsia accounted for the majority of HDP (52.5%) in this study. This is comparable to other studies in Ethiopia; 51.8% in Jimma [10], 60.7% in Mettu [24] but less than the 78% in Addis Ababa [9]. The prevalence of eclampsia in our study was only 2.6% which is comparable to the study in Jimma [10] but much less than the 19% report of eclampsiain Mettu [24], 27.8% in Debrebrhan [28] and Addis Ababa [9]. The decreased proportion of eclampsia in the present study could partly be attributed to early diagnosis of preeclampsia, stringent medical management and termination of pregnancy after ascertaining fetal maturity before sequelae of preeclampsia ensued but still risking fetal prematurity. The increased awareness towards the complication of HDP coupled with the increasing trend in the ANC utilization by the urban residents in the recent years due to massive work by the Ethiopian government [25] might have also contributed for the lower proportion eclampsia in the current urban study.

Majority of the women affected by the hypertensive disorders were nullipara(55.8%) and 91% of mothers had at most 34 years of age. This is similar with the findings of other studies conducted in Ethiopia [10, 24, 28]. However, there was no statistically significant association between age of mothers and severity of hypertensive disorders (*p* = 0.15). In 53.2% of women with HDP, onset of labor was by induction and the likelihood of induced labor was significantly higher with severity of HDP (*p* < 0.001). Almost all inductions were for women with preeclampsia, eclampsia and superimposed preeclampsia; which is less than the finding from studies in Debrebrhan (60.9%) [28], but greater than studies in Jimma(36.6%) [10], and Mettu(44.6%) [24]. The mode of delivery was significantly associated with severity of HDP(*p* = 0.009). The rate of cesarean section was 42.5% and instrumental delivery contributed for 3.9% of all deliveries of mothers with HDP. The current cesarean rate was higher than the study in Jimma (34%) [10], Mettu (16.2%) [24], and Debrebrhan (6.3%) [28]. On the contrary, the rate of instrumental delivery was slightly lower than the study in Jimma (7.8%) [10] and Mettu (6.9%) [24], but much lower than the study in debrebrhan (34.7%) [28]. The presence of appropriate professionals in better number in the urban setting might have contributed to the higher cesarean delivery in the present study area contributing to the increased rate of prematurity. Most of the women (76.3%) with hypertensive disorders had the highest systolic blood pressure record of at least 160 mmHg and 83% had the highest diastolic blood pressure of at least 110 mmHg. This finding is in agreement to the study in Mettu [24].

Headache refractory to the ordinary analgesics was among the chief complaints in 30.8% of all mothers with HDP and in 58.5% of pre-eclamptic and eclamptic mothers combined. This is in agreement with the 48.9% incidence of headache among mothers with severe preeclampsia and superimposed preeclampsia from Debrebrhan [28] and similar studies from other countries; 46.2% in Iran [30], and 42.22% in India [29]. Blurring of vision was observed in 22.5% of subjects with HDP and in 42.7% with pre-eclampsia and eclampsia combined. This is also in agreement with other reports [28–30]. In our study, all the eclamptic mothers had preceding complaints of headache and blurring of vision before they were progressed to develop the abnormal body movement and loss of consciousness. Therefore, early detection and subsequent intervention of severe preeclampsia including termination of the pregnancy could have paramount importance in averting the sequelae. In developed countries pregnancy related acute renal failure (AKI) has decreased, with current estimates are around 1–2.8%, where as in developing countries it is 4.5–15% and responsible for both maternal and fetal morbidity and mortality [31]. Some setups showed an alarmingly high value to a 36% with HDP [32]. Our study indicated the prevalence of AKI (Cr > 1.2 mg/dl) to be 10.9% among mothers with HDP which is relatively higher than the reports in Mettu (6.6%) [24]. This could partly be attributed to the increased prevalence of the HDP in the present study. However, the rate of AKI (10.9%) and thrombocytopenia (7.7%) are less than similar studies in other countries [30]**.**

Preeclampsia/eclampsia is responsible for an estimated 16% of global maternal mortality annually [17] and according to ACOG the HDP account for 17.6% of direct maternal deaths [26]**.** The profound risk of maternal death is much more common in settings where prenatal, intrapartum and postpartum care is not routinely available to pregnant women [8, 17]. Surprisingly, maternal mortality associated with HDP or ICU admission in the present study was non-existent even if the HDP is rampant in the study area. This is uncommon finding even compared to the other Ethiopian report of maternal mortality of 2.5% in Debrebrhan [28], 1.2% in Jimma [10], and also the national cause-specific case fatality rate of 3.6% [8]. It is in agreement to the zero mortality rate of the study in Mettu [24]. This could be attributed partly to the fact that our hospital having improved care facilities, well- staffed with appropriate professionals, giving services for 24 h a day-7 days a week, early presentation of the mothers to the hospital and/or health centers in the catchment area, vibrant referral system to the hospital from the health centers, and stringent interventions in the hospital including the termination of pregnancy might have contribute to no maternal death. WHO study also showed that the availability of basic and comprehensive EmOC 24 h per day, 7 days per week—in conjunction with a functioning referral system—is thought to prevent most maternal deaths with direct causes [33]. The low institutional delivery in the country [25] could be another contributing factor for the low maternal mortality observed in this study.

Hypertensive disorders of pregnancy are known to be associated with a number of perinatal complications which can be measured in terms of prevalence of preterm delivery, low birth weight, low Apgar score, intrauterine growth restriction, the need for resuscitation and/or admission to a neonatal intensive care unit (NICU), and stillbirths. In the present study, there was statistically significant association of preterm delivery with severity of HDP (*p* < 0.001). The rate of preterm delivery was 29.5%, out of which severe pre-eclampsia and eclampsia accounted for over 80% of preterm deliveries. This finding is comparable with the study from Jimma (31.6%) [10] and Mettu (28.1%) [24] but less than reports of 35.4% in Debrebrhan [28], and 48.6% in Addis Ababa [9]. The study also showed increased likelihood of low birth weight with increasing severity of HDP (*p* < 0.001). The rate of low Apgar at the first minute and 5th minute has statistically significant association with the severity of HDP (p < 0.001). This is in agreement with other reports [10, 19, 24]. Preeclampsia-eclampsia can also lead to higher frequency of neonatal respiratory distress, and increased frequency of admission to neonatal intensive care unit [13]. Those infants born small and premature may experience low Apgar, perinatal asphysia, prolonged stays in neonatal intensive care units and often face developmental delays [13]. In our study, the need for resuscitational support was 15%, of which 87% were neonates born to mothers with severe pre-eclampsia and eclampsia. Similarly, 11.5% of neonates required referral to NICU, of which 89% were neonates born to severe pre-eclamptic and eclamptic mothers. The rate of NICU admission is comparable to the study from Jimma(16.4%) [10], but much less than the 40.1% of the Debrebrhan study [28].

The WHO multicountry survey has shown that there were about 3- and 5-fold increased risk of perinatal death in women with preeclampsia and eclampsia, respectively [21]. In Ethiopia, hypertensive disorders of pregnancy account for perinatal mortality rate of 290/1000 total births [23]. There were 4 still births in our study yielding still birth rate of 2.6%. This rate is much less than other studies in Ethiopia; 10.7% in Mettu [24], 27.5% in Jimma [10], 30.8% in Debrebrhan [28]. This contracted prevalence of still birth in Yekatit-12 teaching hospital could be partly due to the extensively coordinated and commendable effort by every stakeholder and the efficient referral system from health centers to the hospital for every woman with the risk factor. The other very important contributing factor why the incidence of still birth is so low in the present study area could be associated with the low prevalence of eclampsia (2.6%) as compared to other studies in Ethiopia [10, 24, 28]. Eclampsia accounts for 5-fold increased risk perinatal death [21]. The limited sample size could also have partly contributed to the low stillbirth rate.

## Conclusions

This study revealed higher prevalence of hypertensive disorders of pregnancy compared to some other similar studies in the country. Such an elevated prevalence can be expected in the tertiary referral hospital recieving cases of complicated pregnancies from very large and wide catchment population. Hypertensive disorders of pregnancy have detrimental feto-maternal outcomes mostly due to severe preeclampsia. There was no maternal mortality encountered in the present study, the rate of eclampsia was relatively low, and the rate of still birth attributable to the hypertensive disorders of pregnancy was also low compared to similar studies done in Ethiopia. Much of the obstetric researches in the past several decades have been directed at finding ways to prevent preeclampsia and eclampsia. However, there is no definitive preventive method for the hypertensive disorders of pregnancy to date apart from termination of pregnancy. Therefore, it is imperative to expand and strengthen the focused antenatal surveillance to early recognize the pregnant women with hypertensive disorders of pregnancy, provide them appropriate care and/or refer to the hospital with better care facilities. Up to date and goal oriented training for lower and middle level health professionals at the health centers and in the Hospitals can further increase their capacity for early detection of high-risk pregnancies, and timely referral to advanced tertiary health facilities.

## Data Availability

The datasets used and/or analyzed during the current study are available from the corresponding author on reasonable request.

## Abbreviations

ACOG: American college of obstetrics and gynecology
AKI: Acute kidney injury
ANC: Antenatal care
APGAR: Appearance, pulse, grimace, activity, respiration
BP: Blood pressure
GA: Gestational age
HDP: Hypertensive disorders of pregnancy
HELLP: Hemolysis, elevated liver enzymes and low platelets
IUGR: Intrauterine growth restriction
NICU: Neonatal intensive care unit
